# Advancements in sacroiliac joint reduction for enhancing lumbosacral pain relief and achieving balanced gait: A literature review

**DOI:** 10.1097/MD.0000000000040350

**Published:** 2024-12-13

**Authors:** Jingjing Zheng, Chen Duan, Chaoyang Ma

**Affiliations:** a Department of Rehabilitation Medicine, The Central Hospital of Wuhan, Tongji Medical College, Huazhong University of Science and Technology, Wuhan, China.

**Keywords:** balance gait, lumbosacral pain, research progress, sacroiliac joint reduction

## Abstract

This article provides a comprehensive review of recent research advancements in sacroiliac joint reduction therapy for addressing lumbosacral pain and gait balance issues, delving into its application efficacy, future outlook, and existing challenges. Current literatures were searched on sacroiliac joint reduction therapy, lumbosacral pain and gait balance disorders using the databases PubMed and Cochrane. There were no restrictions when conducting the literature search with regard to publication date, study language, or study type. Research indicates notable enhancements in various gait parameters, including stride length, gait speed, and cycle uniformity, among patients undergoing sacroiliac joint reduction therapy. These improvements translate into augmented walking stability and a reduced risk of falls. Despite its clinical efficacy, this therapeutic modality encounters several challenges in practical implementation. One major hurdle is the absence of standardized international diagnostic criteria for sacroiliac joint dysfunction, hindering the widespread adoption and standardization of this treatment approach. Further clinical investigations and longitudinal data are imperative to ascertain the long-term efficacy and potential risks associated with this therapy. Future research avenues should prioritize the development of precise diagnostic tools and standardized treatment protocols to enhance the efficacy and safety of sacroiliac joint reduction therapy. Moreover, interdisciplinary collaboration is paramount, leveraging the expertise of physical therapists, rehabilitation specialists, and spine surgeons to offer comprehensive treatment solutions. Sacroiliac joint reduction therapy emerges as a compelling therapeutic option for individuals grappling with lumbosacral pain and gait instability, showcasing significant clinical potential and promising future prospects.

## 1. Introduction

The sacroiliac joint, situated between the spine and pelvis, plays a pivotal role in supporting the upper body’s weight and facilitating strength transmission during movement.^[1]^ Structurally, it comprises the ilium and sacrum interconnected by a complex network of ligaments and muscles. While offering stability, this structure also permits limited mobility to accommodate various body postures and movements. Given its unique position and function, the sacroiliac joint’s health significantly impacts the lower back and lower limb function.^[2,3]^ In clinical practice, lumbosacral pain is a prevalent health concern, with approximately 80% of adults experiencing low back pain at some point. Many instances of lumbosacral pain stem from sacroiliac joint dysfunction. Additionally, sacroiliac joint diseases or dysfunctions can influence an individual’s gait. Gait, the rhythmic motion of walking, is fundamental to daily activities and relies heavily on sacroiliac joint stability.^[4,5]^ Any disruption in joint function can lead to gait instability, thereby impacting overall body movement efficiency and comfort. Consequently, diseases and dysfunctions of the sacroiliac joints and their impact on lumbosacral pain and gait balance have become focal points in sports medicine, rehabilitation, and pain management. In recent years, sacroiliac joint reduction therapy – entailing position adjustment or function enhancement through manipulation or physical therapy – has gained widespread usage to alleviate lumbosacral pain and restore normal gait.^[6]^ This approach not only targets symptom relief but also aims to restore joint function and improve patients’ quality of life.^[6]^ The objective of this review is to analyze recent research advancements concerning how sacroiliac joint reduction therapy can ameliorate lumbosacral pain and gait balance. By synthesizing existing research findings, our aim is to offer evidence-based treatment recommendations for clinicians and enhance treatment efficacy for patients.

## 2. Sacroiliac joint diseases and symptoms

Sacroiliac joint dysfunction, owing to its central location in the human body, profoundly impacts the functionality of the lower back, hips, and lower limbs.^[7]^ The pathological state of this joint not only induces pain but also can lead to a spectrum of dysfunctions, significantly affecting individuals’ quality of life and daily activities.^[7]^ Sacroiliac joint dysfunction can be broadly categorized into 2 types: hypermobility dysfunction and hypomobility dysfunction.^[7]^ Hypermobility dysfunction typically arises from excessive joint laxity, with patients often experiencing sensations of unstable joint positioning and susceptibility to dislocation.^[8–10]^ Additionally, hormonal fluctuations during pregnancy can relax sacroiliac joints and other ligaments, predisposing individuals to such dysfunction. Hypomobility dysfunction, on the other hand, is characterized by stiff joints and limited mobility. This form of impairment is commonly observed in the elderly or individuals with prolonged sedentary lifestyles. Joint stiffness not only restricts range of motion but can also result in persistent pain and discomfort. These symptoms may also stem from conditions like arthritis, chronic inflammation, or aforementioned degenerative changes. During clinical evaluation, meticulous collection of medical history is paramount as the initial step. Physicians inquire about the onset, nature (e.g., dull and sharp pain), specific location, and exacerbating or alleviating factors of the pain. Physical examinations often include the Gaenslen test, a commonly employed method to diagnose sacroiliac joint pain. This test involves stretching 1 knee toward the chest while the other leg is extended and pressed down, often inducing or exacerbating pain at the sacroiliac joint.^[11]^ Tenderness point examinations entail applying pressure to specific areas around the sacroiliac joints to pinpoint the source of pain. The FABER/Patrick test assesses sacroiliac joint pain and flexibility by positioning the patient’s foot on the opposite thigh and applying pressure to the hip to observe any pain or discomfort.^[12]^ Imaging examinations offer valuable insights to confirm diagnoses.^[13]^ X-ray examinations reveal bone alignment and any abnormal indicators such as changes in joint space.^[13]^ CT scans provide detailed images of bones and surrounding soft tissues, particularly useful for diagnosing complex fractures and structural issues.^[6]^ MRI scans are ideal for evaluating soft tissues such as ligaments and muscles, as well as detecting early signs of inflammation.^[7]^

Lumbosacral pain, a common manifestation of sacroiliac joint dysfunction, presents a significant challenge to patients’ well-being and profoundly impacts their daily quality of life.^[14–16]^ Typically localized in the lower spine and pelvic region, this pain exhibits variability and persistence, often influenced by daily activities. It manifests as dull aches, tingling, or burning sensations, frequently radiating from the waist to the buttocks and occasionally extending down the back of the thigh. Such radiation pain arises from nerve compression or irritation around the sacroiliac joint, particularly when the sciatic nerve is involved, leading to what’s known as sciatica. Pain exacerbation is often posture-related; prolonged standing or sitting exacerbates symptoms due to continuous pressure on the sacroiliac joints. Movements like bending, twisting, or lifting heavy objects may also trigger or intensify the pain. Nocturnal pain, a prevalent symptom among lumbosacral pain patients, can worsen due to improper sleeping posture or maintaining the same position for extended periods, significantly impacting sleep quality and next-day mobility.^[17–19]^ Sleep deprivation or poor quality sleep exacerbates pain sensations, creating a detrimental cycle. Muscle tension, particularly in the waist and buttocks, is common in lumbosacral pain patients, restricting range of motion and potentially leading to muscle fatigue and stiffness. Persistent muscle tension reduces blood flow, impairs tissue repair, and exacerbates pain. Consequently, patients often experience functional limitations,^[20]^ hindering daily tasks such as bending, dressing, or driving, consequently affecting work, social engagement, and overall quality of life. Despite the association between lumbosacral pain and increased physical activity, finding a balance between rest and activity is crucial for pain management. Adequate rest alleviates joint stress, while regular, gentle physical activity enhances joint flexibility and muscle strength, thereby reducing pain frequency and intensity.

Gait imbalance stands out as a prevalent complication of sacroiliac joint dysfunction, exerting a significant impact on patients’ daily lives.^[21]^ Characterized by unstable or uncoordinated walking, gait imbalance heightens the risk of falling, particularly among the elderly population.^[22]^ Patients may exhibit inconsistent pace, trembling, or a propensity to stumble while walking. This is often attributed to functional limitations imposed by pain, prompting patients to subconsciously alter their walking patterns to mitigate discomfort or accommodate physical constraints. For instance, if dysfunction in 1 sacroiliac joint causes pain, patients may overly rely on the opposite leg for support and mobility, resulting in an asymmetrical gait. Such asymmetry not only elevates fall risks but can also induce pain and dysfunction in other areas such as the knee joint and lower back. Clinical observation forms the cornerstone of gait imbalance assessment. Physicians or physiotherapists meticulously observe patients’ posture, stride length, frequency, and body symmetry during walking. Additionally, observing activities like standing up, turning, and sitting down unveils issues related to balance and coordination. In more advanced settings, gait laboratory analysis utilizes high-speed cameras and pressure sensors to meticulously record all stages of walking. This analysis yields detailed data on various gait parameters, including foot force distribution, timing of each stage of the walking cycle, and motion trajectories of different body parts. Such data aid in pinpointing specific gait parameters associated with dysfunction and elucidating potential reasons for gait alterations. In clinical settings, simple walking tests like the 10-m walking test are commonly employed to assess walking speed and gait stability. This test requires patients to walk as quickly and safely as possible within a designated distance, while the tester records the time and observes gait stability. Rapid and straightforward, this test furnishes practical insights into patients’ daily walking capabilities.

## 3. Treatment of sacroiliac joint reduction

Sacroiliac joint reduction therapy encompasses a variety of approaches, including manual therapy, physical therapy techniques, and targeted exercise regimens, all aimed at restoring normal joint function, alleviating pain, and enhancing overall motor abilities.^[6]^ Among these methods, manipulation therapy, particularly spinal manipulation therapy, is widely utilized for treating sacroiliac joint dysfunction. This approach involves using hands or specialized tools to adjust the sacroiliac joint with precision and force, thereby optimizing alignment, reducing pain, and restoring natural range of motion. Throughout the treatment process, chiropractors conduct thorough assessments to identify specific issues affecting the sacroiliac joint and surrounding tissues. Patients typically assume a lateral position during treatment, allowing therapists to apply controlled force to the joints through rapid, precise manipulations, promoting subtle joint movement and alleviating tension, pain, while bolstering the body’s self-healing mechanisms. Physical therapy constitutes another cornerstone of sacroiliac joint reduction therapy,^[23,24]^ comprising various manual techniques, mechanical interventions, and electrotherapy modalities. Physiotherapists deploy an array of techniques such as massage, traction, thermotherapy, cryotherapy, and ultrasound to mitigate pain, enhance joint mobility, and boost blood circulation. Exercise therapy within physical therapy is particularly crucial for fortifying muscle strength, enhancing joint stability, and forestalling future injuries. Tailored rehabilitation plans are devised based on individual patient needs, encompassing specific stretching and strengthening exercises, along with balance and coordination training. Customized exercise programs targeting sacroiliac joint dysfunction are integral components of reduction therapy. These exercises aim to bolster the muscles of the waist, buttocks, and abdomen, directly or indirectly supporting and stabilizing the sacroiliac joint. Pelvic tilt exercises, for instance, aid in core muscle strengthening and pelvic realignment. To perform this exercise, patients should lie flat on their backs, bend their knees, and gently tilt their pelvis towards their back by engaging their abdominal muscles. Bridge exercises effectively engage the back and buttock muscles, fortifying pelvic support. Lower limb stretching exercises further enhance thigh and buttock muscle strength, reinforcing pelvic and sacroiliac joint stability. Professional guidance ensures correct execution of these exercises, averting potential injuries stemming from improper posture. Consistent practice and gradual intensity escalation are pivotal for long-term management of sacroiliac joint dysfunction and prevention of future recurrence.

Among the array of treatment modalities available, sacroiliac joint reduction, physical therapy, pharmacotherapy, and behavioral interventions emerge as the most commonly employed methods.^[25]^ Sacroiliac joint reduction primarily targets patients experiencing pain and functional limitations due to joint misalignment or dysfunction. This approach swiftly alleviates pain and enhances joint mechanical function, although the duration of its effects and its universal applicability warrant further investigation. Physical therapy, encompassing exercise therapy and electrical stimulation, offers a noninvasive treatment avenue, gradually bolstering muscle strength and joint stability. However, significant improvement may require more time to manifest. Pharmacotherapy effectively and promptly mitigates pain but fails to address underlying biomechanical issues and may carry side effects. Contemporary medicine increasingly advocates for interdisciplinary treatment approaches to tackle complex musculoskeletal disorders like sacroiliac joint dysfunction. Integrating the expertise of chiropractors, physiotherapists, pain specialists, and mental health professionals facilitates the development of comprehensive treatment plans focusing not only on symptom relief but also on enhancing overall health and quality of life. Future research endeavors may explore innovative interdisciplinary collaboration models and leverage emerging technologies such as virtual reality and wearable devices to further enhance therapeutic efficacy. Enhancing patients’ quality of life stands as a paramount measure of treatment success. In addressing sacroiliac joint dysfunction, attention must extend beyond pain reduction and functional improvement to encompass patients’ psychological well-being, sleep quality, and daily activities. Long-term follow-up studies underscore the importance of continuous treatment and regular evaluation in maintaining therapeutic efficacy and preventing symptom recurrence. Through periodic follow-up visits and tailored treatment plan adjustments, patients can experience ongoing improvement, with treatment methods dynamically adapted to meet evolving needs.

## 4. Effect of sacroiliac joint reduction on lumbosacral pain

Sacroiliac joint reduction therapy, a widely utilized intervention for lumbosacral pain, has gained significant traction in clinical practice.^[26]^ Its primary objective is to alleviate pain and associated symptoms stemming from joint dysfunction by realigning and optimizing the movement of the sacroiliac joint. Numerous studies have investigated the impact of sacroiliac joint reduction on patients with lumbosacral pain.^[27]^ Employing randomized controlled trial designs, the majority of these studies ensure data reliability and validity. Findings consistently demonstrate that sacroiliac joint reduction substantially reduces pain intensity and enhances patients’ functional status and quality of life.^[28]^ From a biomechanical standpoint, manipulation adjustments improve sacroiliac joint alignment and tension, consequently alleviating abnormal pressure distribution within and around the joint.^[29]^ Reduction therapy facilitates local blood circulation, enhances tissue nutrient supply, and eliminates metabolic waste, thereby reducing inflammation and alleviating pain. From a neurophysiological perspective, sacroiliac joint reduction may achieve pain relief by modulating nervous system function.^[30]^ Reset operations stimulate surrounding mechanoreceptors and nerve endings, activating central nervous system pain regulation pathways, thereby diminishing pain perception. This “neuromodulation” effect transiently alleviates the patient’s pain sensation. Specific case studies and clinical trials further corroborate the efficacy of sacroiliac joint reduction in lumbosacral pain treatment. For instance, 1 case study documents a patient experiencing prolonged lumbosacral pain. Following several weeks of sacroiliac joint reduction treatment, significant pain relief was observed, enabling the patient to resume normal activities, leading to improved sleep quality and overall mood.^[31]^ In another clinical trial involving a sizable sample, patients were randomly assigned to receive sacroiliac joint reduction treatment or serve as controls. Posttreatment, the treatment group reported not only a marked decrease in pain intensity but also substantial improvement in daily functioning. These improvements persist during the follow-up period, underscoring the long-term effectiveness of sacroiliac joint reduction therapy.^[32]^

## 5. Effect of sacroiliac joint reduction on gait balance

**Figure 1.**
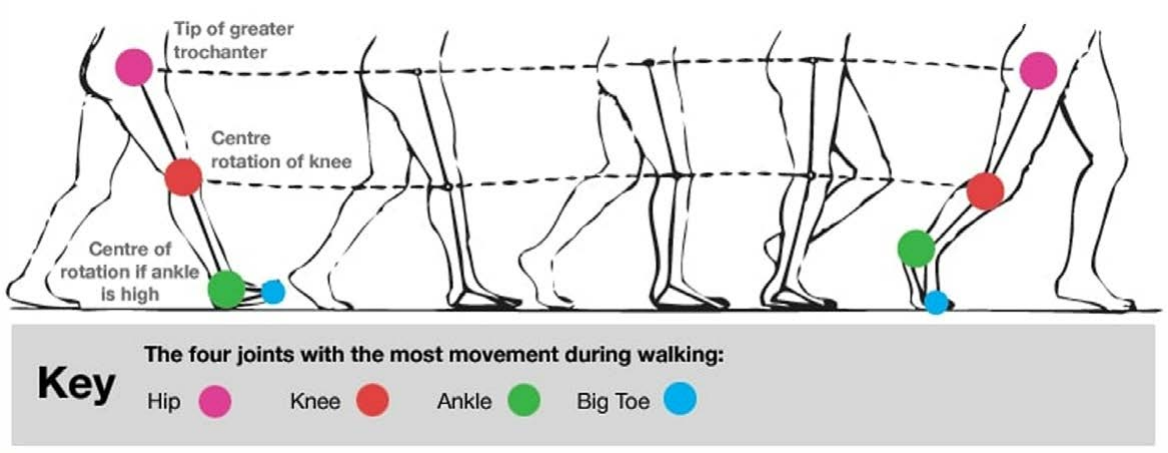
Relationship between gait imbalance and sacroiliac joint dysfunction.

Sacroiliac joint reduction therapy is commonly utilized in clinical settings to ameliorate lumbosacral pain and associated dysfunction, yet its impact on gait balance merits attention.^[26]^ Gait balance denotes individuals’ ability to maintain physical stability while walking, directly influencing walking efficiency and safety.^[33]^ The sacroiliac joint, crucially positioned between the lower limbs and trunk, significantly influences gait balance (Fig. 1).^[21]^

Gait analysis serves as a valuable tool for evaluating the efficacy of sacroiliac joint reduction therapy, providing detailed insights into patients’ gait alterations pre- and posttreatment. This analysis enables clinicians to comprehensively assess the specific impact of sacroiliac joint reduction treatment on patients’ walking abilities by quantifying and recording key parameters such as stride length, pace, gait cycle, and symmetry. Step length and pace serve as fundamental parameters for gauging gait efficiency.^[34]^ Typically, increases in stride length and pace signify enhanced utilization of lower limb strength during walking, often attributed to correct adjustments in sacroiliac joint positioning and function. Following sacroiliac joint reduction, improved joint alignment and pain relief enable patients to take longer strides and walk at a faster pace, thereby enhancing overall walking efficiency. The uniformity of the gait cycle and improvement in gait symmetry represent 2 additional crucial indices of sacroiliac joint reduction treatment efficacy. Gait cycle uniformity pertains to the consistency of left and right leg movements during walking, while gait symmetry refers to the balance of left and right body movements during walking. By rectifying sacroiliac joint dysfunction, reduction therapy facilitates the restoration of normal gait rhythm and symmetrical walking patterns, thereby mitigating gait asymmetry attributed to pain or dysfunction. This enhancement directly contributes to reducing the risk of falls and enhancing walking stability.

Sacroiliac joint reduction therapy exerts a profound influence on patients’ overall balance and stability, constituting a pivotal aspect of its therapeutic efficacy. By rectifying abnormal joint positioning and dysfunction, this treatment modality optimizes strength distribution and mechanical dynamics within the pelvic region. Normalization of sacroiliac joint position and function ensures more balanced lower limb strength distribution during weight-bearing activities like standing and walking, thereby enhancing overall stability. Notably, sacroiliac joint reduction alleviates unnecessary muscle tension and overcompensation resulting from joint instability, crucial for maintaining balance.^[35]^ Clinical research evaluating the efficacy of reduction therapy often includes balance assessments. Results consistently demonstrate significant improvements in patients’ ability to maintain balance while standing on 1 foot and during dynamic walking. These tests simulate real-world challenges encountered in daily life, and the enhanced performance following reduction therapy underscores patients’ improved ability to cope with fall risks.

**Figure 2.**
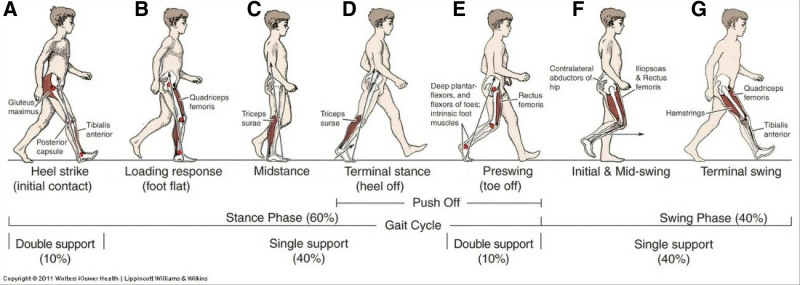
Effects of sacroiliac joint reduction from a biomechanical perspective.

From a biomechanical perspective (Fig. 2), exploring the impact of sacroiliac joint reduction reveals how this treatment optimizes strength distribution and movement coordination within the human body, thereby enhancing patients’ exercise efficiency. Positioned between the spine and lower limbs, the sacroiliac joint bears the dual responsibilities of weight transfer and impact absorption. Maintaining joint stability is crucial for ensuring consistent force transmission, thereby enhancing efficiency and safety during walking and standing. Dysfunctional sacroiliac joints disrupt this continuity, resulting in irregular gait and coordination.^[36]^ Hence, the objective of joint reduction is to restore normal anatomical positioning and biomechanical function, consequently improving overall dynamic stability. Several notable biomechanical changes ensue following sacroiliac joint reduction. Correct joint alignment reduces pelvic area asymmetry, facilitating more even load distribution during walking or standing. Joint position adjustment directly impacts muscle tension and activation patterns. Studies indicate that following reduction treatment, activity levels of muscles overactive due to joint instability – such as those in the waist and lower limbs – decrease. This reduction in muscle overuse lowers energy consumption, enhances walking economy, and significantly reduces fatigue levels during prolonged activities.

## 6. Research progress and practical challenges

Sacroiliac joint reduction has demonstrated remarkable efficacy in clinical settings, particularly in alleviating lumbosacral pain and enhancing gait balance. Through precise adjustment of the sacroiliac joint position, this treatment directly influences patients’ biomechanical mechanisms and pain perception. Numerous studies employing gait analysis technology have shown significant improvements in walking stability, stride length, pace, and gait cycle uniformity among patients postreduction treatment. These enhancements not only facilitate easier walking in daily life but also effectively mitigate the risk of falls due to unstable gait, thereby enhancing overall patient quality of life. Sacroiliac joint reduction therapy significantly contributes to improving walking economy.^[37]^ By optimizing joint biomechanical function, it reduces unnecessary muscle strain and energy consumption, thus markedly diminishing fatigue levels during prolonged walking or standing. This is particularly beneficial for elderly patients or individuals requiring prolonged standing in their professions. Despite its manifold advantages, sacroiliac joint reduction therapy encounters several challenges in clinical application.^[38]^ Foremost among these is the lack of a standardized diagnostic criterion for sacroiliac joint dysfunction, hampering treatment popularization and standardization. Current diagnosis relies heavily on clinician experience and symptom interpretation, lacking clear and quantifiable diagnostic tools, which may introduce subjectivity and diagnostic errors. Furthermore, there is a paucity of scientific data supporting the long-term effects and safety of reduction therapy. While most studies focus on short-term effects, long-term follow-up studies posttreatment remain relatively sparse. Particularly, potential side effects or long-term consequences of treatment, such as excessive joint relaxation or instability, warrant deeper understanding through extensive long-term clinical trials. Individual patient differences significantly influence treatment efficacy, necessitating comprehensive individual evaluation before treatment implementation. Factors such as age, sex, lifestyle, and specific health status may impact therapeutic outcomes. Therefore, personalized treatment plans tailored to individual needs, with adjustments in frequency, intensity, and specific techniques, are pivotal for optimizing treatment efficacy. Addressing these challenges, future research should prioritize the development of standardized diagnostic tools to enhance the accuracy and efficiency of sacroiliac joint dysfunction diagnosis. Additionally, large-scale long-term clinical trials are imperative to comprehensively study the long-term effects and potential risks of reduction therapy, providing a more scientific treatment basis for clinical practice. Future treatment methods should emphasize interdisciplinary collaboration, integrating the expertise of physiotherapists, chiropractors, pain specialists, and mental health professionals to deliver comprehensive treatment plans. Leveraging high-tech auxiliary methods such as virtual reality and biofeedback technology can enhance treatment interaction and patient participation, further optimizing treatment efficacy.

## 7. Summary

Sacroiliac joint reduction therapy stands out as an effective approach for addressing both lumbosacral pain and gait imbalance. This treatment modality, involving manipulation to adjust the sacroiliac joint position, directly contributes to pain alleviation and motor function enhancement. Gait analysis outcomes consistently reveal notable improvements post-sacroiliac joint reduction, including increased stride length, enhanced gait speed, optimized gait cycle, and improved gait symmetry. These enhancements not only bolster walking stability and efficiency but also mitigate fatigue during prolonged walking or standing. Despite its positive outcomes, sacroiliac joint reduction therapy encounters challenges in clinical implementation. The absence of a unified diagnostic standard for sacroiliac joint dysfunction impedes treatment popularization and efficacy to some extent. Additionally, more extensive clinical trials and studies are imperative to validate the long-term efficacy and safety of reduction therapy. Furthermore, recognizing individual patient differences is crucial, necessitating the development of more personalized treatment protocols to cater to specific patient needs. Future research endeavors should prioritize the development of standardized diagnostic tools to enhance diagnostic accuracy and efficiency. Moreover, large-scale, long-term clinical trials are essential to comprehensively explore the long-term efficacy and potential side effects of reduction therapy. Exploring interdisciplinary treatment approaches to integrate expertise from diverse fields can further enhance treatment efficacy, providing patients with comprehensive and efficient treatment regimens. Taken together, sacroiliac joint reduction therapy represents a valuable treatment option for patients grappling with lumbosacral pain and gait instability, significantly enhancing their quality of life. With ongoing research advancements and accumulated clinical experience, the future application prospects of this treatment method are promising.

## Author contributions

**Conceptualization:** Chen Duan.

**Data curation:** Jingjing Zheng, Chen Duan, Chaoyang Ma.

**Formal analysis:** Jingjing Zheng, Chen Duan, Chaoyang Ma.

**Investigation:** Jingjing Zheng, Chaoyang Ma.

**Writing – original draft:** Jingjing Zheng, Chaoyang Ma.

**Writing – review & editing:** Chen Duan.

## Abbreviations:

None
